# A Rare Case of Takayasu Arteritis Presenting With Syncope and Elevated Inflammatory Markers in an Elderly Male With Learning Disability

**DOI:** 10.7759/cureus.87320

**Published:** 2025-07-05

**Authors:** Fayyas Ahamed, Mohamed Abdulmajeed, Ali Haider, Julee William, Kenneth Lee

**Affiliations:** 1 General Internal Medicine, Luton And Dunstable University Hospital, Luton, GBR; 2 General Internal Medicine, Luton and Dunstable University Hospital, Luton, GBR

**Keywords:** cardiology corner, cardiovascular disease, internal medicine, large vessel vasculitis, takayasu disease

## Abstract

Takayasu arteritis (TAK) is a rare large-vessel vasculitis predominantly affecting young females. We present a unique case of a 77-year-old male with learning disability, presenting with constitutional symptoms, syncope, and elevated inflammatory markers. Imaging revealed circumferential thickening of the aorta and arch vessels, consistent with TAK. This case underscores the importance of considering large-vessel vasculitis even in elderly patients, as well as the diagnostic challenges in individuals with cognitive impairment. The patient responded well to corticosteroid therapy.

## Introduction

Takayasu arteritis (TAK) is a chronic inflammatory granulomatous disease of large arteries, primarily affecting the aorta and its major branches. It typically presents in women under 40 years of age, especially in Asian populations, and is exceedingly rare in elderly males [1]. The clinical presentation is heterogeneous and may include systemic symptoms such as fever, fatigue, syncope, and elevated acute-phase reactants [2]. Because of its nonspecific features, TAK is often misdiagnosed or diagnosed late.

Diagnosis is established through clinical features, laboratory markers of inflammation, and imaging, typically CT angiography, MR angiography, or PET-CT [3]. In resource-constrained or behaviorally challenging patients, Doppler ultrasonography has shown value in detecting arterial wall thickening and halo signs [4]. Early recognition and prompt immunosuppression are critical to prevent irreversible vascular damage [5].

We present an unusual case of TAK in an elderly male with learning disability, emphasizing diagnostic challenges and therapeutic response.

## Case presentation

A 77-year-old male with a background of type 2 diabetes mellitus, hypertension, prior transient ischemic attack (TIA), and a learning disability presented with a two-day history of a dry cough, dizziness, and a witnessed episode of syncope lasting approximately 1-2 minutes. Following the syncopal event, the patient was noted to be more confused than his baseline cognitive status.

He denied associated symptoms such as fever, chest pain, vomiting, or loss of bladder/bowel continence. On initial assessment, he was alert but mildly disoriented, with a Glasgow Coma Scale (GCS) score of 14/15 and an Abbreviated Mental Test (AMT) score of 1/4.

The patient was vitally stable with a blood pressure of 108/56 mmHg, heart rate of 98 beats/min, respiratory rate of 20 breaths/min, SpO₂ 94% on 2 L/min oxygen, and temperature of 37.2°C.

Chest auscultation revealed occasional bilateral crepitations. Cardiovascular, abdominal, and focused neurological examinations were otherwise unremarkable. A thorough vascular examination was performed, which revealed palpable and symmetrical peripheral pulses in all four limbs without any evidence of pulse asymmetry. No bruits were auscultated over the carotid, subclavian, abdominal, or femoral arteries. Blood pressure measurements in both arms were comparable, with no significant inter-arm difference noted. There were no clinical signs of limb ischemia, such as pallor, coolness, or delayed capillary refill.

Initial laboratory investigations revealed elevated inflammatory markers, normocytic anemia, and thrombocytosis. Imaging with a chest X-ray showed bibasal atelectasis without focal consolidation. CT of the head demonstrated no acute pathology. The patient was empirically started on intravenous co-amoxiclav and oral clarithromycin for presumed community-acquired pneumonia, which was later escalated to piperacillin-tazobactam, and subsequently to meropenem, due to persistently elevated C-reactive protein (CRP) levels (Table 1). Although initial imaging did not show clear consolidation, the patient presented with elevated inflammatory markers, mild respiratory symptoms, and bibasal atelectasis on chest X-ray, which raised suspicion of an atypical or early pneumonia, particularly given his advanced age, cognitive impairment, and increased risk of aspiration. Persistently elevated C-reactive protein (>120 mg/L) and thrombocytosis despite initial antibiotics raised concern for possible resistant or occult infection (e.g., aspiration pneumonia, empyema, or extrapulmonary source). Given the high risk of morbidity from untreated sepsis in an elderly, vulnerable patient, clinicians opted for a stepwise escalation of antibiotics while concurrently investigating non-infectious causes of inflammation.

**Table 1:** Laboratory test results CRP, C-reactive protein; WBC, white blood cells; Hb, hemoglobin; ESR, erythrocyte sedimentation rate; IgG, immunoglobulin G; IgA, immunoglobulin A

Test	Result	Reference Range	Notes
CRP	136 mg/L (initial)	<5 mg/L	Elevated; >120 mg/L on repeat readings
WBC	9.9 × 10⁹/L	4.0–11.0 × 10⁹/L	Within normal limits
Hb	96 g/L	130–170 g/L (male) / 120–150 g/L (female)	Low
Platelets	508 × 10⁹/L	150–400 × 10⁹/L	Elevated
ESR	83 mm/hr	<20 mm/hr (age- and sex-dependent)	Elevated
IgG	26.79 g/L	7.0–16.0 g/L	Elevated
IgA	4.89 g/L	0.7–4.0 g/L	Elevated
Folate	3.3 nmol/L	>7.0 nmol/L	Low
Serum protein electrophoresis	Minor monoclonal band	No monoclonal bands	Pattern consistent with chronic inflammation

The lack of clinical and radiological improvement despite broad-spectrum coverage ultimately led to reconsideration of the diagnosis and evaluation for vasculitis. Additional investigations revealed significantly elevated erythrocyte sedimentation rate (ESR) and immunoglobulin levels, low folate, and a serum protein electrophoresis pattern consistent with chronic inflammation and a minor monoclonal band.

**Figure 1:**
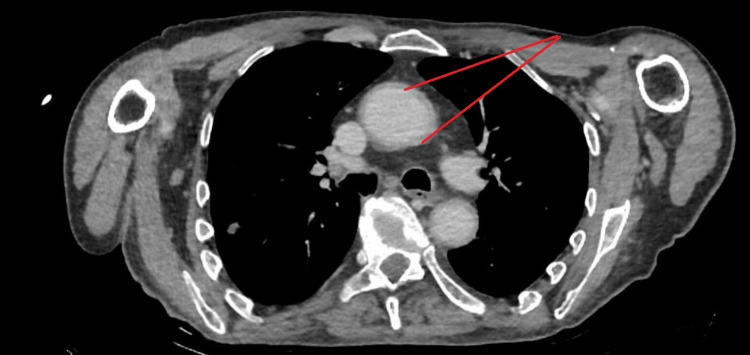
CT aortogram Red arrows point to circumferential aorta thickening

A contrast-enhanced CT of the thorax, abdomen, and pelvis demonstrated circumferential thickening of the aortic wall, most prominent at the aortic arch, extending into the ascending and descending thoracic aorta. Additionally, the origins of the major arch branches - including the brachiocephalic artery, left common carotid artery, and left subclavian artery - showed wall thickening and luminal narrowing, consistent with large-vessel vasculitis (LVV). No significant involvement of the abdominal aorta or its major branches was observed (Figure 1), raising suspicion for LVV. PET-CT was attempted for further evaluation but was unsuccessful due to patient agitation.

**Figure 2:**
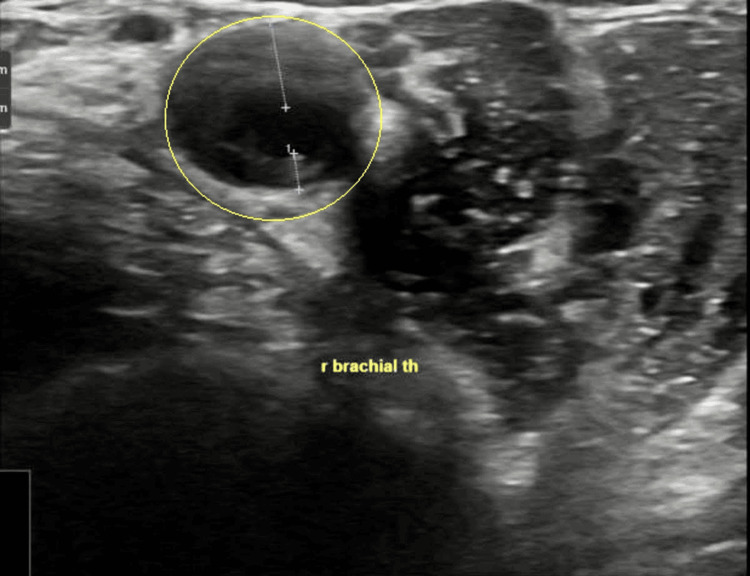
Ultrasound Doppler of the brachial artery Yellow circle points to the brachial artery halo sign

Subsequent ultrasound Doppler studies of the brachial, temporal, and axillary arteries revealed mildly increased intima-media thickness (IMT) in the temporal arteries, along with significantly increased IMT in both the axillary and brachial arteries. Notably, bilateral halo signs were observed in the brachial arteries (Figure 2).

Given the imaging and laboratory findings suggestive of LVV, particularly involving the aortic arch and major branches, the patient was commenced on oral prednisolone 40 mg daily. The initial dose was selected based on standard first-line management guidelines for suspected giant cell arteritis (GCA) and related LVV in elderly patients, aiming to induce rapid suppression of vascular inflammation and prevent further ischemic complications such as vision loss or cerebrovascular events.

A tapering schedule was planned over several weeks, contingent on clinical response and inflammatory marker trends. Within five days of corticosteroid initiation, the patient demonstrated significant improvement in both cognitive status and general well-being. By 20/05/25, CRP had reduced from >120 mg/L to 19 mg/L, indicating a favorable early response to therapy.

Additional supportive management included close monitoring of blood glucose levels due to the patient’s pre-existing type 2 diabetes and the heightened risk of steroid-induced hyperglycemia. Daily functional and neurological assessments were performed to detect any evolving deficits or further vascular events. Gastroprotection with a proton-pump inhibitor was initiated to reduce the risk of steroid-induced gastritis or ulceration. Bone protection measures, including calcium and vitamin D supplementation, were started given the patient’s age and the anticipated prolonged course of corticosteroid therapy. For long-term management, a referral to rheumatology was arranged for outpatient follow-up, with consideration of steroid-sparing agents such as methotrexate or tocilizumab in the event of relapse, steroid resistance, or toxicity.

## Discussion

TAK presents a diagnostic and therapeutic challenge due to its rarity, heterogeneous presentation, and overlap with other inflammatory or infectious diseases. In this case, the diagnosis of TAK was supported by the modified Ishikawa criteria, which incorporate clinical features (e.g., age at onset, constitutional symptoms), elevated inflammatory markers, and imaging evidence of large-vessel inflammation [1]. In our patient, these criteria were met through the presence of systemic symptoms, raised acute-phase reactants, and imaging findings of aortic and major branch involvement, while alternative diagnoses were excluded. The patient’s cognitive impairment further complicated the clinical picture. Traditionally, TAK affects women under 40, and atypical demographics, such as elderly men, are at a risk of misdiagnosis or delayed diagnosis due to low initial clinical suspicion [1].

Syncope, as observed in our patient, is an uncommon but documented manifestation of TAK. It likely results from compromised cerebral perfusion due to stenosis or inflammation of the aortic arch and carotid arteries. This emphasizes the importance of considering LVV in elderly patients presenting with unexplained syncope, particularly when conventional causes are excluded [2].

Another diagnostic clue in this patient was the persistence of systemic inflammation despite appropriate antimicrobial therapy. This non-resolving inflammatory state, marked by elevated CRP, ESR, and immunoglobulins, suggested an underlying autoimmune or inflammatory pathology. While serum protein electrophoresis revealed a minor monoclonal band, consistent with age-related changes or chronic inflammation, it was not diagnostic. The importance of integrating imaging early in such cases cannot be overstated.

Imaging remains the cornerstone of TAK diagnosis. In this case, CT angiography revealed circumferential aortic thickening - a classic radiologic hallmark of TAK [3]. However, in patients with agitation, cognitive impairment, or contrast limitations, advanced imaging such as PET-CT or MR angiography may be impractical. Here, Doppler ultrasonography provided decisive evidence. The halo sign and increased IMT in the axillary and temporal arteries are now well-established surrogate markers for active arteritis. Recent studies support the diagnostic sensitivity and specificity of these signs in both TAK and GCA [4].

While GCA was considered in the differential diagnosis - particularly given the patient’s age and elevated inflammatory markers - several clinical and imaging findings favored TAK. The vascular involvement was predominantly in the aortic arch and its major branches rather than the cranial arteries typically affected in GCA. The patient had no symptoms of cranial ischemia, such as headache, scalp tenderness, jaw claudication, or visual disturbances, which are characteristic of GCA. Ultrasound showed halo signs in the brachial rather than temporal artery, and CT imaging demonstrated circumferential thickening of the aortic wall and arch vessels, findings more consistent with TAK. Other potential mimics, such as infectious aortitis, were deemed unlikely due to negative cultures and lack of infectious source. IgG4-related disease was not supported by clinical or serologic findings, and lymphoma was considered but ruled out based on imaging and clinical course. These considerations collectively supported the diagnosis of TAK in this atypical demographic.

Management of TAK is typically initiated with high-dose glucocorticoids. Our patient’s rapid response to corticosteroids aligns with current evidence that early treatment can control inflammation and reduce vascular damage. However, relapse rates remain high with corticosteroids alone. Approximately 50% of patients eventually require additional immunosuppressive therapy, such as methotrexate, azathioprine, or biologics such as tocilizumab, particularly in refractory or relapsing disease [5]. Although our patient responded well to steroids, longitudinal monitoring through imaging and laboratory markers is critical to detect subclinical disease progression.

Additionally, the case underscores the challenges of managing systemic autoimmune diseases in patients with learning disabilities or cognitive decline. These individuals may be unable to reliably communicate symptoms or side effects, necessitating close coordination with caregivers and multidisciplinary teams. Early involvement of rheumatology, geriatrics, and primary care is crucial to ensure comprehensive care and adherence to monitoring protocols [6].

Lastly, this case illustrates the broader clinical lesson of anchoring bias, where clinicians may prematurely conclude a diagnosis based on common conditions like infection in the elderly. Maintaining a broad differential and re-evaluating initial assumptions in light of non-response to therapy is essential. As life expectancy increases and imaging technologies advance, clinicians are likely to encounter more atypical presentations of historically age-restricted diseases such as TAK.

## Conclusions

TAK, although typically seen in younger females, can present atypically in elderly males and should be considered when faced with persistent systemic inflammation, raised inflammatory markers, and vague constitutional symptoms such as fatigue, syncope, or confusion, especially in patients with cognitive impairment or limited communication.

When advanced imaging such as PET-CT is unavailable or impractical, Doppler ultrasonography serves as a valuable diagnostic tool, offering non-invasive detection of hallmark features such as halo signs and increased IMT. Prompt initiation of corticosteroid therapy can lead to marked clinical improvement and may significantly alter the disease course, even in medically complex individuals.
